# Longitudinal integration of microbiota and metabolomics reveals (poly)phenols-driven gut ecosystem dynamics

**DOI:** 10.3389/fnut.2026.1858875

**Published:** 2026-07-13

**Authors:** Catarina J. G. Pinto, Carlos Pita, Rafael Carecho, Natasa Loncarevic, María Ángeles Ávila-Gálvez, Juan Carlos Espín, Antonio González-Sarrías, Joana Séneca, David Berry, Cláudia Nunes dos Santos

**Affiliations:** 1 NOVA Institute for Medical Systems Biology (NIMSB), Universidade Nova de Lisboa, Lisboa, Portugal; 2 iNOVA4Health, Programme in Translational Medicine, Lisboa, Portugal; 3 iNOVA4Health, NOVA Medical School, Faculdade de Ciências Médicas, NMS|FCM, Universidade Nova de Lisboa, Lisboa, Portugal; 4 IBMC, Instituto de Biologia Molecular e Celular, Universidade do Porto, Porto, Portugal; 5 I3S, Instituto de Investigação e Inovação em Saúde, Universidade do Porto, Porto, Portugal; 6 Laboratory of Food and Health, Group of Quality, Safety, and Bioactivity of Plant Foods, CEBAS-CSIC, Murcia, Spain; 7 Group of Quality, Safety, and Bioactivity of Plant Foods, CEBAS-CSIC, Murcia, Spain; 8 Joint Microbiome Facility of the Medical University of Vienna and the University of Vienna, Vienna, Austria; 9 Department of Microbiology and Ecosystem Science, Centre for Microbiology and Environmental Systems Science, University of Vienna, Vienna, Austria; 10 iBET, Instituto de Biologia Experimental e Tecnológica, Oeiras, Portugal

**Keywords:** dietary (poly)phenols, diet-microbe-metabolite axis, fecal metabolomics, gut microbiota remodeling, microbial temporal dynamics

## Abstract

The gut microbiota and its “theatre of activity” (the collective pool of metabolites and signaling molecules) function as a plastic interface that responds to nutritional inputs. Dietary (poly)phenols are key bioactive components of berries and are increasingly recognized for their capacity to modulate gut microbiota composition and metabolic activity while being biotransformed by microbiota. However, the temporal dynamics of this interaction remain poorly resolved, as most studies rely on cross-sectional or endpoint-only designs. Here, we conducted a longitudinal multi-omic study to map the co-evolution of the fecal microbiota and the metabolome during a sustained consumption of a berry-enriched diet. Using a murine model, fecal samples were collected at baseline, mid-intervention (day 21) and endpoint (day 42). These samples were analyzed by 16S rRNA gene sequencing and untargeted metabolomics to distinguish transient perturbations from stabilized ecosystem reorganization. Two-stage ecosystem reconfiguration was observed concurrent with the intervention: an early, high-magnitude reconfiguration phase (D0 - D21), followed by a period of stabilization (D21 - D42). The initial phase was characterized by the enrichment of the tryptophan metabolic pathway and significant taxonomic shifts, including the proliferation of Lachnospiraceae and Oscillospiraceae alongside the depletion of Prevotellaceae and Akkermansiaceae. Conversely, the stabilization phase was defined by the emergence of tyrosine-derived aromatic signatures and the recovery of Muribaculaceae. Integrated Procrustes analysis confirmed that the strongest coordination between the microbiota and metabolome occurred during the first 21 days, suggesting that microbial composition and metabolic output co-evolve most dynamically during the initial exposure to berry (poly)phenols. We conclude that dietary exposure to berries is associated with a rapid, coordinated restructuring of the gut ecosystem that stabilizes over time, emphasizing that longitudinal multi-omic designs are essential to capture the transient metabolic nodes and stable configurations that define the diet-microbe-metabolite loop.

## 1 Introduction

The gut microbiota is a central mediator of host metabolic health, functioning as a plastic interface that responds to nutritional inputs (1). Diet is a primary driver of microbial community assembly and functional output, with macronutrients and phytochemicals shaping the competitive fitness of resident taxa (2). Beyond compositional changes, diet modulates the “theatre of activity,” which corresponds to the collective pool of metabolites, enzymes, and signaling molecules that determines the ecosystem’s functional state (3). Time-resolved studies show that shifts in both composition and metabolic output can occur within days, highlighting that the gut ecosystem is not static but undergoes dynamic transitions between functional configurations (4–6).

Berries are rich sources of (poly)phenols, including anthocyanins, proanthocyanidins, ellagitannins, and phenolic acids, which have been consistently linked to cardiometabolic, inflammatory, and cognitive benefits (7–9). These clinical effects have stimulated interest in the biological mechanisms underlying (poly)phenol-mediated health outcomes. A defining feature of berry (poly)phenols is their reciprocal interaction with the gut microbiota, forming a diet-microbe-metabolite loop central to understanding their physiological effects and inter-individual variability in response (10, 11). Due to their limited absorption in the upper gastrointestinal tract, (poly)phenols reach the colon largely intact (12–15) and undergo extensive microbial biotransformation into smaller, often more bioactive metabolites (10, 15–17). In turn, (poly)phenols and their metabolites modulate microbial composition and predicted functional capacity, enriching some taxa and suppressing others (11, 18, 19). These bidirectional processes mean that microbial composition and metabolic output co-adapt in response to berry intake rather than shifting in isolation.

Despite these established links, the temporal structure of (poly)phenol-microbiota interactions remains under-resolved at the systems level. Most studies rely on cross-sectional or endpoint-only sampling, which obscures early versus late responses and misses transient microbial-metabolite couplings (4, 20, 21). Methodological work emphasizes that longitudinal designs must account for within-subject dependence and compositionality to avoid biased trajectory inferences (22). In order to track the co-evolution of taxa and metabolites during and after dietary exposure, repeated sampling and multi-omics analysis are needed.

Because the microbiome comprises both the community and its functional readouts, pairing microbiota profiling with metabolomics is required to resolve functional redundancy and detect metabolic reconfiguration not reflected at the taxonomic level (3, 23). Multi-omic approaches, including congruency analyses, latent-variable models, and timepoint-specific networks, reveal coordinated microbiota-metabolite modules that single-layer analyses overlook (24). These considerations are especially relevant for dietary interventions, where rapid remodeling cautions against endpoint-only designs (4).

To address these gaps, we conducted a 42-day longitudinal multi-omic study to map how a berry-enriched diet remodels the murine fecal ecosystem across baseline, mid-intervention, and endpoint. Because our model utilized young mice (6 weeks of age at baseline), tracking long-term dietary adaptations requires accounting for the timeline of natural microbiome maturation. Longitudinal studies under unperturbed standard chow conditions have demonstrated that the murine gut microbiota naturally completes its post-weaning ecological succession and consolidates into a highly constant, stable adult homeostatic configuration by approximately 5.5 to 6 weeks of age, with no significant changes up to 12 weeks of age (25, 26). By establishing our experimental baseline precisely at 6 weeks of age, we targeted this expected developmental stabilization. We hypothesized that a sustained berry-derived (poly)phenol intervention would override this background homeostatic window, driving a distinct multi-omic restructuring phase that would eventually transition into a novel, diet-adapted functional plateau. Specifically, we aimed to characterize the temporal dynamics of microbiota and metabolome interactions and to determine whether berry intervention induces a transient shift or a persistent, coordinated reconfiguration of the “theatre of activity” within the gut ecosystem.

## 2 Materials and methods

### 2.1 Animals, experimental design and sampling

For the dietary intervention, twenty-four 6-week-old male C57BL/6 J mice were used, corresponding to 12 cage-level biological units. Animals were purchased from Charles River Laboratories (France) at 4 weeks of age, housed in pairs, therefore, the cage was considered the experimental unit throughout the study, as indicated by “n.” Following arrival, mice underwent 1 week of quarantine and 1 additional week of acclimatization. Animals were then fed for 6 weeks with a berry-enriched diet rich in (poly)phenols, consisting of standard chow (Teklad 2018, Envigo, USA) supplemented with 8% freeze-dried berry mixture composed of equal proportions of blueberry (*Vaccinium* spp., variety Star), blackberry (*Rubus* spp., variety Loch Ness), and raspberry (*Rubus idaeus* L., variety Kweli), produced in Odemira region, Portugal. Fresh food was provided daily, and food intake was monitored throughout the intervention. Fecal pellets were collected on days 0, 21, and 42, with two pellets per cage placed into separate DNase- and RNase-free tubes for downstream analyses and stored at −80 °C until use. All animal procedures were approved by the NOVA Medical School Ethics Committee and by the Portuguese national authority Direção-Geral de Alimentação e Veterinária (reference no. 0421/000/000/2021), in accordance with Council Directive 86/609/EEC.

### 2.2 Microbial DNA extraction and 16S analysis

Bacterial DNA extraction was performed with a QIAamp DNA Stool Mini Kit (Qiagen #50951604) from cage-level fecal samples, according to the manufacturer’s instructions, with an additional mechanical disruption step, as described by others (27). At the end of the extraction, DNA was eluted with 30 μL of DNase, RNase-free water (Fisher Scientific # 10295243). Columns were incubated for 5 min at RT and centrifuged at full speed for 1 min to elute the DNA. DNA was quantified and purity was assessed using the Nanodrop 2000 (Thermo Scientific). The V4 hypervariable region of the bacterial and archaeal 16S rRNA gene was amplified using primers 515F/806R (28, 29). DNA sequencing and raw data processing were performed at the Joint Microbiome Facility of the Medical University of Vienna as described previously (30) and sequenced on an Illumina MiSeq (2 × 300 bp). Amplicon pools were extracted from the raw sequencing data using the FASTQ workflow in BaseSpace (Illumina) with default parameters. Raw data processing was performed as described previously (30). Demultiplexing was performed using the Python package demultiplex (31), allowing one mismatch for barcodes and two mismatches for linkers and primers. Amplicon sequence variants (ASVs) were inferred using the DADA2 R package v1.42 (32) applying the recommended workflow (33). FASTQ reads 1 and 2 were trimmed at 220 nt and 150 nt with allowed expected errors of 2. ASV sequences were subsequently classified using DADA2 and the SILVA database SSU Ref NR 99 release 138.1 (34, 35) using a confidence threshold of 0.5 to maximize classification depth. ASVs without classification or classified as eukaryotes, mitochondria, or chloroplasts, as well as well-known buffer contaminations, were removed.

### 2.3 Untargeted metabolomic analysis by UPLC-ESI-QTOF-MS of mouse fecal samples

For untargeted metabolomics, 20 mg of each sample were extracted with 1 mL of methanol/chloroform/water (30:10:60, v/v/v) to recover primarily polar metabolites. Samples were vortexed for 1 min and centrifuged at 14,000 × g for 10 min at 4 °C. The resulting supernatants were collected and evaporated to dryness under vacuum using a SpeedVac concentrator. Dried extracts were then reconstituted in 200 μL of methanol containing 0.1% formic acid prior to LC–MS analysis.

The analyses were carried out using an Agilent 1,290 Infinity LC system coupled to an Agilent 6,550 iFunnel Accurate-Mass QTOF mass spectrometer (Agilent Technologies, Santa Clara, CA, USA) equipped with a dual electrospray ionization source (ESI-Jet Stream Technology). The system allowed continuous mass correction through the simultaneous introduction of a reference mass solution, ensuring high mass accuracy throughout the analytical sequence. Data acquisition was performed using MassHunter Workstation software (version B.08.00, Agilent).

Chromatographic separation was performed on a Poroshell 120 EC reverse-phase column (3.0 × 100 mm, 2.7 μm; Agilent Technologies), maintained at 30 °C. The injection volume was 1 μL, and the chromatographic run was carried out at a flow rate of 0.5 mL/min. The mobile phases consisted of water acidified with 0.1% formic acid (solvent A) and acetonitrile acidified with 0.1% formic acid (solvent B). Chromatographic separation was achieved using the following gradient program: 0–3 min, 5–18% B; 3–10 min, 18–50% B; 10–13 min, 50–90% B; 13–14 min, 90–5% B; followed by re-equilibration at 5% B until 22 min.

The mass spectrometer was operated in negative ionization mode only, as this approach provides enhanced sensitivity for several metabolite classes expected to be modulated by berry-derived polyphenols, including phenolic acids, microbial-derived catabolites, organic acids, and other acidic polar metabolites. Therefore, the analytical workflow was optimized to maximize the detection of these biologically relevant compounds according to the objectives of the study.

TOF spectra were acquired over a mass range of *m/z* 50–1,100, at an acquisition rate of 1.5 spectra/s, corresponding to 666.7 ms per spectrum, with 5,484 transients per spectrum. The operating parameters of the ion source were set as follows: gas temperature, 280 °C; drying gas flow, 9 L/min; nebulizer pressure, 45 psi; sheath gas temperature, 400 °C; sheath gas flow, 12 L/min; capillary voltage, 3,500 V; nozzle voltage, 500 V; fragmentor voltage, 100 V; skimmer voltage, 65 V; and octopole radiofrequency voltage, 750 V. External calibration of the instrument was performed at the beginning of each batch using the manufacturer’s Tuning Mix. Internal mass calibration was applied during acquisition using reference ions atm/z 112.9855 and m/z 1033.9881 in negative polarity.

To monitor system performance and support analytical reliability, a quality control (QC) sample was included in the analytical batch. This QC injection was used to assess retention time consistency, signal response, and mass accuracy under the selected experimental conditions. In addition, methanol blank injections were introduced at different points in the sequence to monitor potential carry-over effects, background contamination, and signal memory from previously injected samples. The inclusion of multiple methanol blanks also allowed verification of the cleanliness of the chromatographic system and contributed to the reliability of the analytical data.

The acquired LC–MS untargeted data files were converted to *.mzXML format using the ProteWizard MSconvert tool (v 3.0.24074–2,118,430) (36) and subsequently pre-processed with the open-source software MZmine (v 3.9.0) (37), including peak detection, retention time correction, peak matching, and peak filling. Peak detection was performed in three steps: (i) mass detection with noise value = 10,000 and retention time range = 0.8 to 21 min; (ii) chromatogram builder with minimum time spa*n =* 0.2 min, minimum height = 20,000, and *m/z* tolerance = 0.005 Da or 15 ppm; (iii) deconvolution with peak width = 0.05–1.0 min and noise = 10,000. Retention time was corrected using *m/z* tolerance = 0.005 Da or 15 ppm, retention time tolerance = 1.0 min, and minimum standard intensity = 1.0E6. Peak matching among samples was performed with the Join aligner using *m/z* tolerance = 0.005 Da or 15 ppm (weight for *m/z* = 1), retention time tolerance = 0.2 min (weight for retention time = 0.9), and mobility weight = 1. Gap filling was applied using the peak finder method with retention time correction, with intensity tolerance = 30%, *m/z* tolerance = 0.005 Da or 15 ppm, and retention time tolerance = 0.3 min.

The metabolomics data have been deposited to MetaboLights (38) repository with the study identifier MTBLS14779.

### 2.4 Statistical analysis and data visualization

All statistical analyses were performed using the cage as the experimental and biological unit. Thus, observations corresponded to cage-level samples rather than individual animals. Statistical analyses and data visualization were performed using a combination of bioinformatics platforms and R packages, including RStudio (R v4.1.2), GraphPad Prism (v10.3.0), MetaboAnalyst 6.0 (accessed March 2026), MicrobiomeAnalyst 2.0 (accessed March 2026), and OmicsAnalyst 2.0 (accessed March 2026) for multi-omic integration.

For untargeted LC–MS data, one cage-level sample at D0 and one cage-level sample at D21 were excluded due to lack of sample, resulting in 11 cage-level samples at D0 and D21 and 12 cage-level samples at D42 being included for analysis. Mass spectral features were filtered using default settings to remove low-variance and near-zero abundance signals. Data were normalized to the reference QC sample, log10-transformed, and auto-scaled prior to downstream analyses. To account for the longitudinal design and within-cage correlation across D0, D21, and D42, differential abundance analysis was performed in R using the *limma* package (v3.50.3). Cage-specific effects were modeled using the duplicateCorrelation function, and empirical Bayes moderated t-tests were used to identify significantly altered features. *p*-values were adjusted for multiple testing using the Benjamini-Hochberg false discovery rate (FDR). Untargeted metabolomic analyses were performed using LC–MS feature extraction without MS/MS acquisition. Therefore, metabolite annotation was limited to putative feature matching based on accurate mass. Pathway inference was performed in MetaboAnalyst 6.0 using the mummichog algorithm (v2.0) against the *Mus musculus* KEGG library with a significance cutoff of *p <* 0.05. No confirmation with authentic standards or fragmentation spectra was performed. Consequently, metabolite annotations and pathway associations should be interpreted as hypothesis-generating and consistent with low-confidence putative annotations according to Metabolomics Standards Initiative (MSI) guidelines.

The 16S rRNA gene sequencing data were processed in MicrobiomeAnalyst 2.0. Two cages were excluded because their baseline (D0) samples did not yield an acceptable number of identified features after data processing and quality filtering. To maintain the longitudinal paired structure of the dataset, the corresponding D21 and D42 samples from these cages were also removed. Consequently, although 12 cages were initially included, the final 16S analysis was performed on 10 cage-level biological units across all timepoints. Feature filtering was applied using default parameters, and relative abundance tables at family levels were obtained. Alpha diversity was evaluated using the Chao1 and Shannon indices. Predicted microbial functional profiles were inferred using Tax4Fun2 and mapped to the KEGG library, providing inference-based estimates of functionality.

Global structural variation in the metabolome and microbiota was assessed by principal coordinates analysis (PCoA) using the *vegan* package (v2.6–4). Euclidean distances were calculated for the log-transformed and scaled metabolomic data, whereas Bray-Curtis dissimilarities were used for the microbial compositional data. To test for longitudinal differences while accounting for the paired repeated-measures design, paired PERMANOVA was performed using the adonis2 function in vegan with 999 permutations restricted within cage blocks. Homogeneity of multivariate dispersion was assessed using the betadisper and permutest functions to ensure that differences were not driven by dispersion effects.

Coordination between the fecal metabolome and microbiota was evaluated using Procrustes rotation (PROTEST) and Mantel tests implemented in vegan, based on Euclidean and Bray-Curtis distance matrices, respectively. Resulting *p*-values were adjusted using FDR. Integrated multi-omic correlation networks were generated in OmicsAnalyst using DIABLO for supervised multi-omic integration and feature selection, and Spearman’s rank correlation to identify significant associations between microbial families and metabolomic features (|r| > 0.6, *p <* 0.05).

Data visualization, including the generation of row-z-scored heatmaps for *limma*-selected significant features, was performed using the R packages *stats* and *pheatmap* (v. 1.0.12). Ordination plots were produced using *ggplot2* (v3.5.0). Additional statistical analyses and graphical representations were generated in GraphPad Prism. Statistical significance was defined as *p < 0.05, **p < 0.01, and ***p < 0.001.

## 3 Results

### 3.1 Longitudinal impact of a berry-enriched diet on fecal metabolome

Mice were fed a berry-enriched diet *ad libitum* for 42 days. Supplementation of the standard chow with the berry mixture did not alter proximate composition, namely carbohydrate, protein, lipid, or fiber (Supplementary Table S1). Phenolic profiling of both diets confirmed the expected increase in (poly)phenol diversity in the supplemented diet (Supplementary Tables S2, S3).

**Figure 1:**
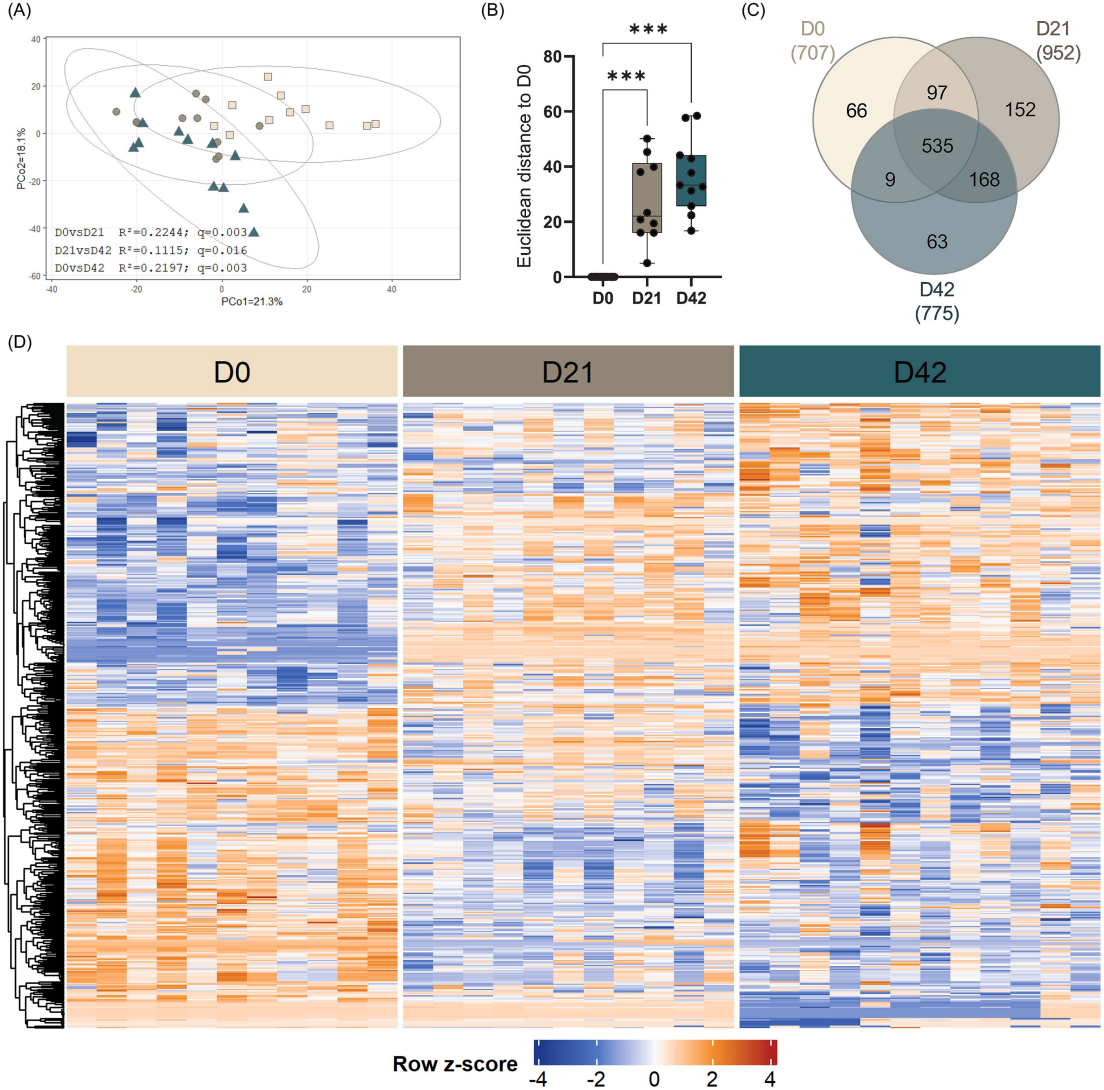
Longitudinal impact of a berry-enriched diet on the murine fecal metabolome. **(A)** Principal Coordinates Analysis (PCoA) based on a Euclidean distance of untargeted metabolomic profiles at D0 (beige squares), D21 (brown circles), and D42 (dark cyan triangles). Each point represents an individual cage-level sample (*n =* 11 for D0 and D21; *n =* 12 for D42) sampled longitudinally. Statistical significance was assessed using permutational multivariate analysis of variance (PERMANOVA) followed by FDR multiple comparisons test (*q* < 0.05). **(B)** Intra-individual Euclidean distance from each cage at D0 baseline in PCoA space. Boxplots represent the spread of dissimilarity for each cage on D21 and D42 and show the median and interquartile range, with whiskers indicating the minimum and maximum values (*n =* 11 for D0 and D21; *n =* 12 for D42). Statistical significance was assessed using a mixed-effect analysis with the Geisser–Greenhouse correction, followed by Sidak’s multiple comparisons test (***p < 0.001). **(C)** Venn diagram illustrating overlap of metabolomic features detected via untargeted LC–MS across timepoints. Values in parentheses indicate the total number of features detected on each day, highlighting both shared and time-specific unique features on D0, D21 and D42. **(D)** Heatmap of the 494 differentially abundant features (FDR-adjusted q < 0.05) identified using limma repeated-measures analysis. Values are presented as row-wise Z-scores of normalized peak areas, grouped using complete linkage hierarchical clustering based on Euclidean distances. Samples (columns) are ordered chronologically (D0, D21, D42) without clustering to visualize longitudinal progression.

To assess the longitudinal variations in the overall fecal metabolome following the introduction of a (poly)phenol-rich diet, untargeted LC–MS analysis was performed at day 0 (D0; prior to dietary intervention), day 21 (D21), and day 42 (D42). Principal Coordinates Analysis combined with paired PERMANOVA (Figure 1A) revealed a clear temporal separation of metabolomic profiles. Significant differences in metabolomic composition were observed between D0 and D21 (*R^2^* = 0.2244 and *q* = 0.003), D21 and D42 (*R^2^* = 0.1115 and *q* = 0.016), and D0 and D42 (*R^2^* = 0.2197 and *q* = 0.003). To evaluate intra-individual metabolic shifts, Euclidean distances from the D0 baseline were calculated for each cage. A significant increase in dissimilarity by D21 (Figure 1B), which persisted without further significant deviation through D42, indicated a sustained shift from the baseline configuration over the course of the study. A total of 1,090 metabolomic features were detected across all samples. Of these, 535 (49.1%) constituted a core metabolome present at all timepoints, while 66, 152, and 63 features were unique to D0, D21, and D42, respectively (Figure 1C). Longitudinal analysis using limma repeated measures identified 494 features that were differentially abundant between at least two timepoints, indicating dynamic metabolic remodeling (Figure 1D). Hierarchical clustering of these features revealed distinct temporal patterns of metabolite abundance, including clusters that increased or decreased at specific stages of the temporal trajectory (Figure 1D). D0 samples formed two distinct clusters, while D21 and D42 displayed more closely related abundance profiles, characterized by smaller sub-clusters differentiating the two timepoints. Together, these data show that continuous intake of a berry-enriched diet is strongly associated with a substantial and time-dependent restructuring of the fecal metabolome, characterized by an initial shift followed by a plateau phase by day 42.

### 3.2 Longitudinal impact of a berry-enriched diet on microbiota

**Figure 2:**
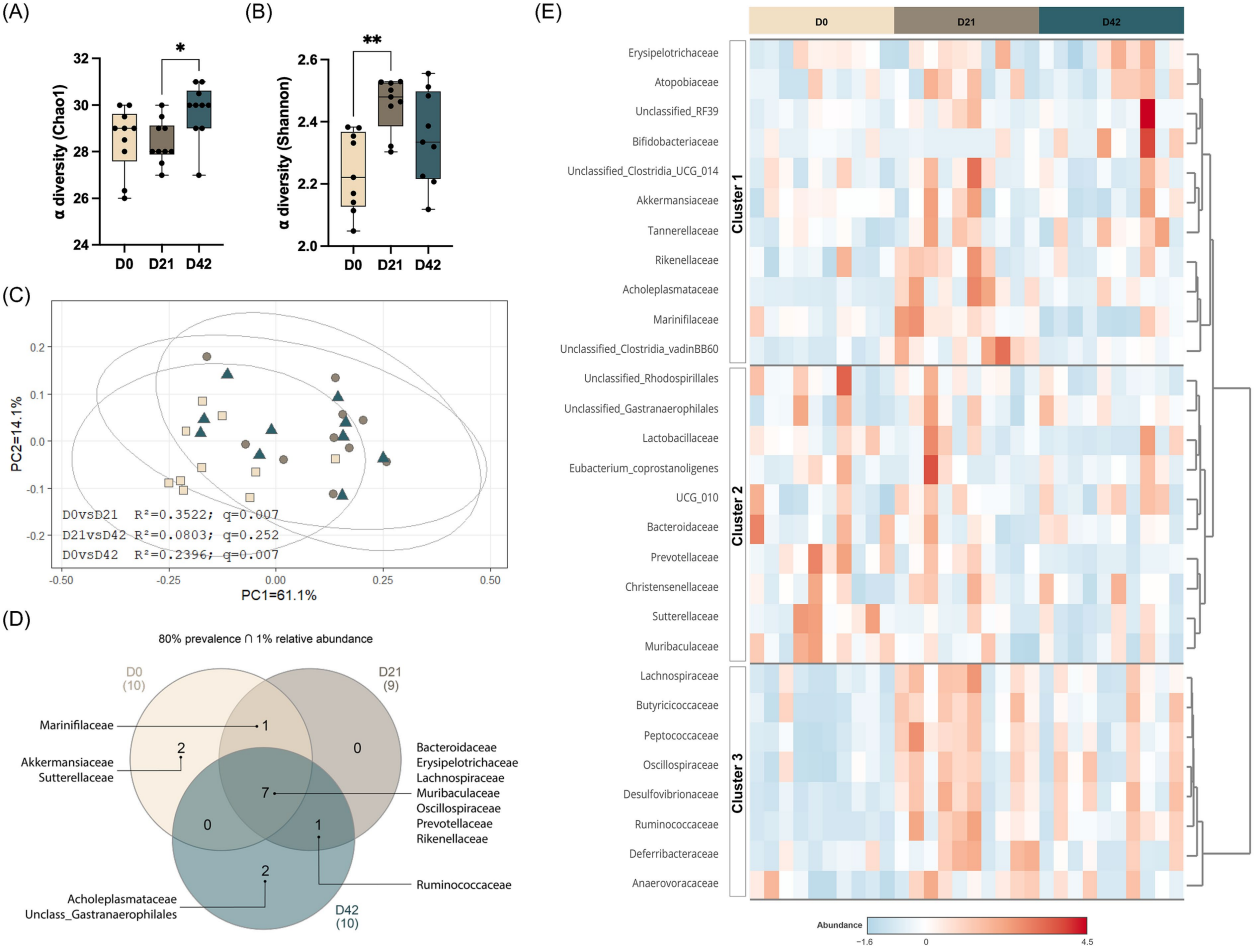
Longitudinal impact of a berry-enriched diet on the murine fecal microbiota. **(A,B)** Alpha diversity estimated by the Chao1 index **(A)**, and Shannon index **(B)** at Day 0 (D0), Day 21 (D21), and Day 42 (D42). Boxplots show the median and interquartile range, with whiskers indicating the minimum and maximum values (*n =* 10). Statistical significance was assessed using paired one-way ANOVA followed by Tukey’s multiple comparisons test (*p < 0.05, **p < 0.01, *p < 0.001). **(C)** Principal coordinates analysis (PCoA) based on a Bray–Curtis dissimilarity matrix of family-level microbiota profiles at D0 (beige squares), D21 (brown circles), and D42 (dark cyan triangles). Each point represents a single cage sampled longitudinally (*n =* 10). Pairwise differences between timepoints were assessed by paired PERMANOVA, and corresponding *q*-values are shown. **(D)** Venn diagram illustrating the shared core microbiota at the family level across D0, D21, and D42, using a cutoff of 80% prevalence and ≥1% relative abundance. **(E)** Heatmap of family-level relative abundance across D0, D21, and D42, with hierarchical clustering of bacterial families performed using Euclidean distance and Ward’s clustering algorithm. Three major clusters with distinct temporal abundance patterns were identified.

To study the longitudinal variations in gut microbial ecology following the introduction of the berry-enriched diet, a longitudinal analysis of the fecal 16S rRNA gene profiles was performed. Alpha-diversity analysis using the Chao1 Index at the family level shows an increase in the number of families between D21 and D42 (Figure 2A). Conversely, alpha-diversity using the Shannon Index showed an increase in microbial diversity between D0 and D21, which is maintained through D42 (Figure 2B). These shifts indicate modifications in both microbial richness and evenness concurrent with the longitudinal berry supplementation trajectory, suggesting greater community complexity. Beta-diversity analysis using a Bray–Curtis dissimilarity and paired PERMANOVA analysis (Figure 2C) showed that the microbial composition on D21 (*R^2^* = 0.3522; *q* = 0.007) and D42 (*R^2^* = 0.2396; *q* = 0.007) differ significantly from the baseline. In contrast, no significant differences were observed between D21 and D42 (*R^2^* = 0.0803; *q* = 0.252), suggesting that microbial community structure entered a phase of relative stabilization compared to the higher-magnitude initial shift.

At the family level, core microbiota analysis showed that the study period was accompanied by a time-dependent restructuring of the shared bacterial community (Figure 2D). Seven families were consistently present across all timepoints, based on a threshold of 80% prevalence and ≥1% relative abundance: Bacteroidaceae, Erysipelotrichaceae, Lachnospiraceae, Muribaculaceae, Oscillospiraceae, Prevotellaceae, and Rikenellaceae. In contrast, some families were restricted to specific stages of the temporal tracking, with Akkermansiaceae and Sutterellaceae being above the threshold only at D0, and Acholeplasmataceae together with unclassified Gastranaerophilales only present in the core at D42. Other families showed transitional patterns, with Marinifilaceae shared between D0 and D21, and Ruminococcaceae shared between D21 and D42.

These results suggest the presence of both stable and dynamic microbial components during the intervention. Finally, hierarchical clustering of family-level abundance profiles revealed three major clusters with distinct temporal trajectories across the dietary intervention (Figure 2E). This pattern indicates that the berry-enriched diet was associated with a remodeling of the fecal microbial community over time, with the most pronounced shift occurring between baseline and the post-intervention timepoints, while D21 and D42 retained a more similar global configuration.

Together, these findings indicate that while a stable core community was maintained throughout the intervention, the longitudinal profile was characterized by time-dependent gains and losses of specific bacterial families, reflecting a dynamic but partially stabilized ecological reorganization.

### 3.3 Metabolome and microbiota composition shifts between days 0 and 21

**Figure 3:**
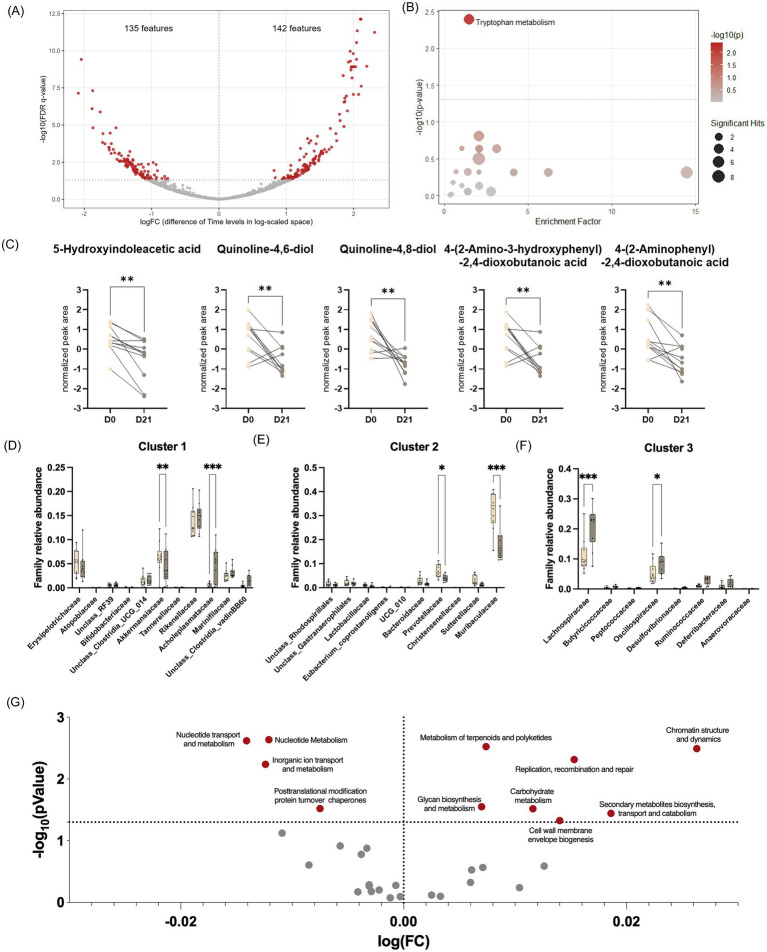
Multi-omic characterization of the gut ecosystem shift from Day 0 to Day 21. **(A)** Volcano plot of fecal metabolome (D21 vs. D0). Differential abundance was assessed using linear mixed-effects models (limma) with intra-subject correlation to account for repeated measures. Features meeting the threshold for statistical significance (FDR-adjusted *p <* 0.05) are highlighted in red. Non-significant features are shown in grey. The total number of significantly depleted (left) and enriched (right) features are annotated at the top of the respective quadrants. **(B)** Mummichog-based pathway enrichment analysis for features differing between D0 and D21. Bubble size reflects the number of significant metabolic features contributing to each pathway, and color corresponds to -log10(*q*-value). **(C)** Pairwise comparison of normalized peak areas at D0 and D21 for mass features associated with the tryptophan metabolism pathway. Feature annotations are putative and based on m/z matching. Statistical significance was assessed via paired t-test or Wilcoxon (**p < 0.01). **(D–F)** Relative abundance of microbial families at D0 (beige) and D21 (brown) (*n =* 10), grouped into three clusters derived from hierarchical clustering of bacterial families, resulting **(D)** Cluster 1, **(E)** Cluster 2 and **(F)** Cluster 3. Statistical significance was assessed using paired one-way ANOVA followed by Tukey’s multiple comparisons test (*p < 0.05, **p < 0.01, *p < 0.001). **(G)** Volcano plot of predicted microbial metabolic pathways (inferred using Tax4Fun2). Red points indicate significantly altered pathways (*p <* 0.05). Functional profiles are inferred from 16S rRNA gene data and should be interpreted as predictions. Statistical significance was assessed using paired multiple t-tests with Holm–Šídák correction for multiple comparisons (*p < 0.05, **p < 0.01, *p < 0.001).

The introduction of a berry-enriched diet was characterized by significant alterations in both the metabolome and microbiota between D0 and D21. Untargeted metabolomics revealed a substantial reshaping of the fecal metabolome, with 25.4% of detected features being differentially abundant (*q* < 0.05), of which 12.4% decreased, and 13% increased at D21 compared to baseline (Figure 3A).

Pathway enrichment analysis using Mummichog and the *Mus musculus* KEGG library enabled functional inference directly from high-resolution mass features without prior metabolite identification highlighting tryptophan metabolism as the top pathway associated with the metabolic shift between D0 and D21 (Figure 3B and Supplementary Table S4). Consistent with this trend, normalized peak areas of mass features contributing to the tryptophan metabolism pathway were significantly reduced at D21 (Figure 3C), including 5-hydroxyindoleacetic acid, quinolin-4,6-diol, quinolin-4,8-diol, and two amino-phenyl-dioxobutanoic acid derivatives.

Microbial community shifts at the family level were also evaluated at the same timepoints. Microbial families’ relative abundances were examined in the context of the previously identified clustering structure (Figure 2E). Within Cluster 1, Akkermansiaceae decreased, while Acholeplasmataceae increased from D0 to D21 (Figure 3D). In Cluster 2, the families contributing most prominently to the observed temporal shift belonged predominantly to the phylum Bacteroidota and showed reduced abundance between D0 and D21 (Figure 3E). In Cluster 3, as also observed in Cluster 2, the families exhibiting the most prominent longitudinal variations were the most abundant within the cluster, both belonging to the phylum Bacillota and increased from D0 to D21 (Figure 3F).

To examine the predicted functional profiles of these taxonomic shifts, microbial metabolic pathway profiles were predicted using Tax4Fun2. This analysis revealed significant differences in the inferred microbial functional profile between D0 and D21 (Figure 3G). Several pathways were significantly enriched or depleted at D21 (*p <* 0.05). Pathways related to nucleotide transport and metabolism, nucleotide metabolism, inorganic ion transport and metabolism, and post-translational modification, protein turnover, and chaperones were reduced at D21 relative to baseline. By contrast, pathways associated with metabolism of terpenoids and polyketides, glycan biosynthesis and metabolism, carbohydrate metabolism, cell wall/membrane/envelope biogenesis, replication, recombination and repair, secondary metabolites biosynthesis, transport and catabolism, and chromatin structure and dynamics were increased at D21. These results suggest an early diet-associated shift in the predicted functional capacity of the gut microbiota.

Taken together, these multi-omic analyses demonstrate that the initial 21 days of berry supplementation are associated with coordinated changes in metabolite profiles, bacterial community structure, and predicted microbial metabolic potential, highlighting a tightly linked host-microbe-metabolic response during the early phase of the intervention.

### 3.4 Stabilization of metabolome and microbiota profiles between days 21 and 42

The introduction of the berry-enriched diet was associated with substantial changes in the microbial community and metabolome during the initial phase of the intervention (between D0 and D21). To assess whether additional remodeling occurred during the later phase of the dietary intervention, we compared fecal metabolomic and microbiome profiles between D21 and D42.

Pairwise PERMANOVA analysis indicated that metabolomic profiles between D21 and D42 remained significantly different (*R^2^* = 0.1115, *q* = 0.016; see Figure 1A). However, limma analysis between those two timepoints showed fewer differentially abundant features between D21 and D42, compared to D0 and D21 (Supplementary Figure S1A vs. Figure 3A). Only 8.5% of features were differentially present, of which 4.8% less abundant in D42 compared to D21, and 3.7% were more abundant in D42 compared to D21. These results indicate a marked attenuation of metabolic changes during the later phase of the intervention. Consistent with this pattern, the pathway enrichment analysis using Mummichog and the *Mus musculus* KEGG library did not identify significantly altered pathways, suggesting a relative stabilization and a marked deceleration in metabolic remodeling (Supplementary Figure S1B and Supplementary Table S5).

At the microbiota level, only minor compositional changes were observed. Within Cluster 1, Acholeplasmataceae significantly decreased on D42 compared to D21 (Supplementary Figure S1C). In Cluster 2, only the Muribaculaceae family increased in abundance on D42 compared to D21 (Supplementary Figure S1D). Cluster 3 presents no statistical differences between D21 and D42 (Supplementary Figure S1E). The inferred functional profiles using Tax4Fun2 also showed no significant differences between D21 and D42 (Supplementary Figure S1F), similarly to what was observed at the metabolome level.

Overall, the comparison between D21 and D42 reveals that the major metabolomic and microbiota shifts observed during the study occurred primarily during the first 21 days, followed by more subtle and targeted adjustments during the subsequent 3 weeks.

### 3.5 Long-term multi-omic shifts in the gut ecosystem from day 0 to day 42

To determine the long-term variations within the gut ecosystem following the introduction of the berry-enriched diet, we compared the fecal metabolomic and microbiota profiles between baseline (D0) and the end of the intervention (D42).

**Figure 4:**
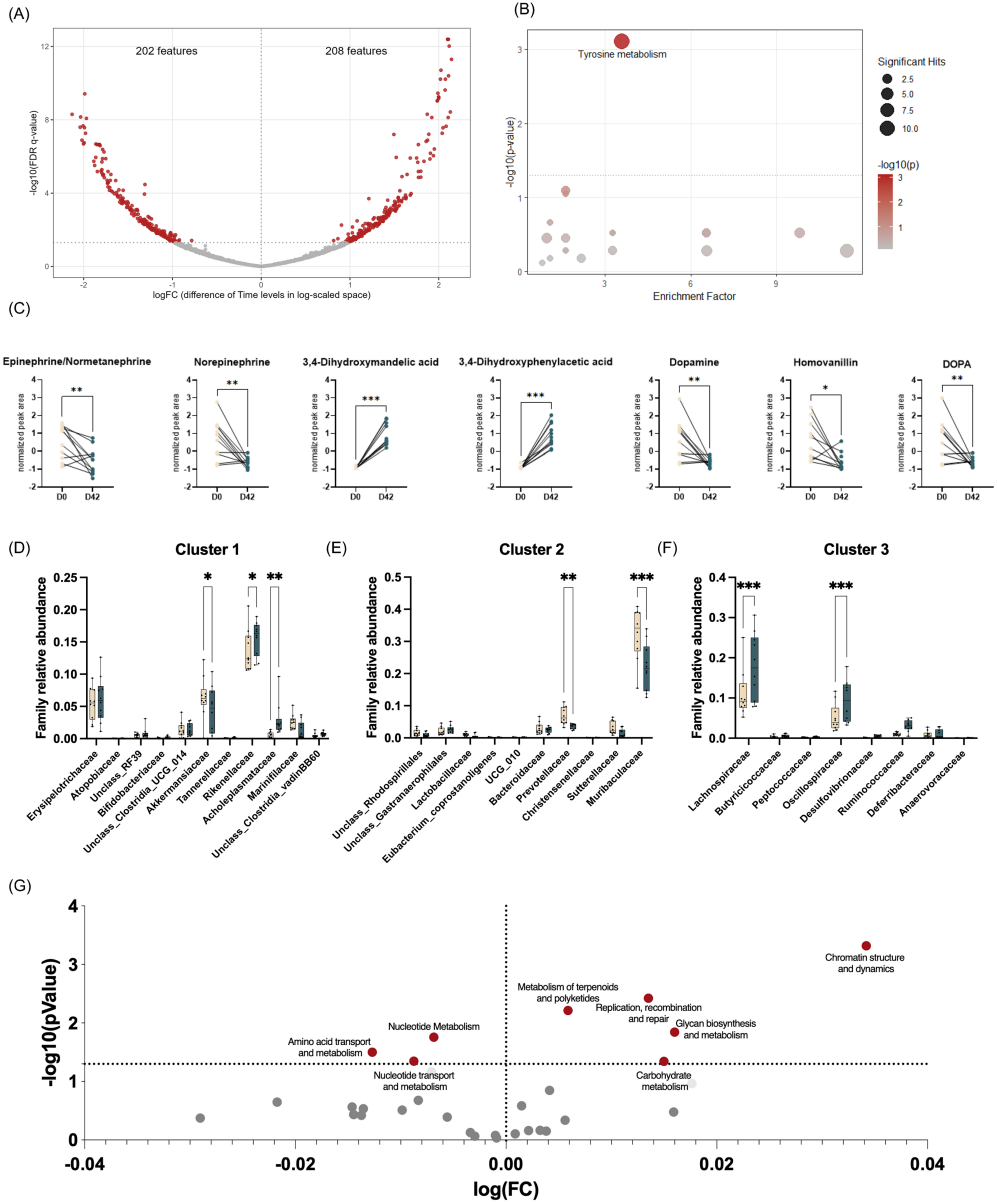
Multi-omic characterization of the gut ecosystem shift from Day 0 to Day 42. **(A)** Volcano plot of fecal metabolomic features (D42 vs. D0). Differential abundance was assessed using linear mixed-effects models (limma) with intra-subject correlation to account for repeated measures. Features meeting the threshold for statistical significance (FDR-adjusted *p <* 0.05) are highlighted in red. Non-significant features are shown in grey. The total number of significantly depleted (left) and enriched (right) features are annotated at the top of the respective quadrants. **(B)** Mummichog-based pathway enrichment analysis using the KEGG *Mus musculus* pathway library. Bubble size indicates the number of significant features mapping to each pathway, and color denotes −log10(*q*-value). **(C)** Pairwise comparison of normalized peak areas at D0 and D42 for putatively identified metabolites based on the Mummichog enrichment pathways and KEGG library. Statistical significance was assessed via paired t-test or Wilcoxon (*p < 0.05, **p < 0.01, ***p < 0.001). **(D–F)** Relative abundance of microbial families at Day 0 (beige) and Day 42 (dark cyan) (*n =* 10), grouped into three clusters identified by hierarchical clustering of bacterial families, resulting **(D)** Cluster 1, **(E)** Cluster 2 and **(F)** Cluster 3. Statistical significance was assessed using paired one-way ANOVA followed by Tukey’s multiple comparisons test (*p < 0.05, **p < 0.01, *p < 0 0.001). **(G)** Volcano plot of predicted microbial metabolic pathways (inferred using Tax4Fun2). Red points indicate significantly altered pathways (*p <* 0.05). Functional profiles are inferred from 16S rRNA gene data and should be interpreted as predictions. Statistical significance was assessed using paired multiple t-tests with Holm-Šídák correction for multiple comparisons (*p < 0.05, **p < 0.01, *p < 0.001).

At the metabolomic level, limma analysis revealed that 37.6% of detected features were significantly different, of which 18.5% were less abundant in D42 than in D0, and 19.1% were more abundant in D42 compared to D0 (Figure 4A), indicating broad metabolic adjustments over the six-week dietary period.

Pathway enrichment analysis using Mummichog and the KEGG *Mus musculus* library showed tyrosine metabolism as the top-enriched pathway, driven by multiple significant metabolite hits (Figure 4B and Supplementary Table S6). Consistent with this enrichment, paired comparisons of putatively identified metabolites showed significant temporal differences across several tyrosine-related compounds, including epinephrine/normetanephrine, normetanephrine, 3,4-dihydroxybenzaldehyde, 3,4-dihydroxyphenylacetic acid, dopamine, homovanillin, and DOPA (Figure 4C). Among these metabolites, features putatively matching 3,4-dihydroxymandelic acid and 3,4-dihydroxyphenylacetic acid showed a significant increase in the normalized peak areas between D0 and D42, whereas the remaining features showed reduced intensities over time (Figure 4C). These findings highlight a substantial shift in catecholamine-associated metabolic pathways during the intervention.

Microbial community composition also underwent pronounced restructuring between D0 and D42. Within Cluster 1, Akkermansiaceae decreased, whereas Rikenellaceae and Acholeplasmataceae increased from D0 to D42 (Figure 4D). In Cluster 2, the families contributing most prominently to the temporal shift belonged predominantly to the phylum Bacteroidota and displayed reduced abundance between D0 and D42 (Figure 4E). In Cluster 3, the most abundant families, also the most abundant within the cluster, belonged to the phylum Bacillota and increased from D0 to D42 (Figure 4F).

Finally, predicted functional profiling analysis revealed significant differences between D0 and D42 (Figure 4G). Tax4Fun2-inferred pathways related to amino acid transport and metabolism, nucleotide transport and metabolism, and nucleotide metabolism were decreased at D42 relative to baseline. By contrast, pathways associated with metabolism of terpenoids and polyketides, replication, recombination and repair, glycan biosynthesis and metabolism, carbohydrate metabolism, and chromatin structure and dynamics showed higher predicted representation at D42. These findings suggest that the study period was characterized by a sustained remodeling of the predicted functional profile of the gut microbiota throughout the intervention, with a broad predicted shift in functional potential of the microbial community that parallels the observed metabolomic alterations.

Overall, sustained berry supplementation was associated with widespread and coordinated shifts in metabolite profiles, bacterial composition, and predicted microbial functional capacity, reflecting a robust reconfiguration of the gut ecosystem.

### 3.6 Coordinated longitudinal shifts in the taxonomic and metabolic landscape

To assess whether the gut microbiota and the fecal metabolome co-varied in a coordinated manner across the study period, we performed an integrated two-omics analysis.

**Figure 5:**
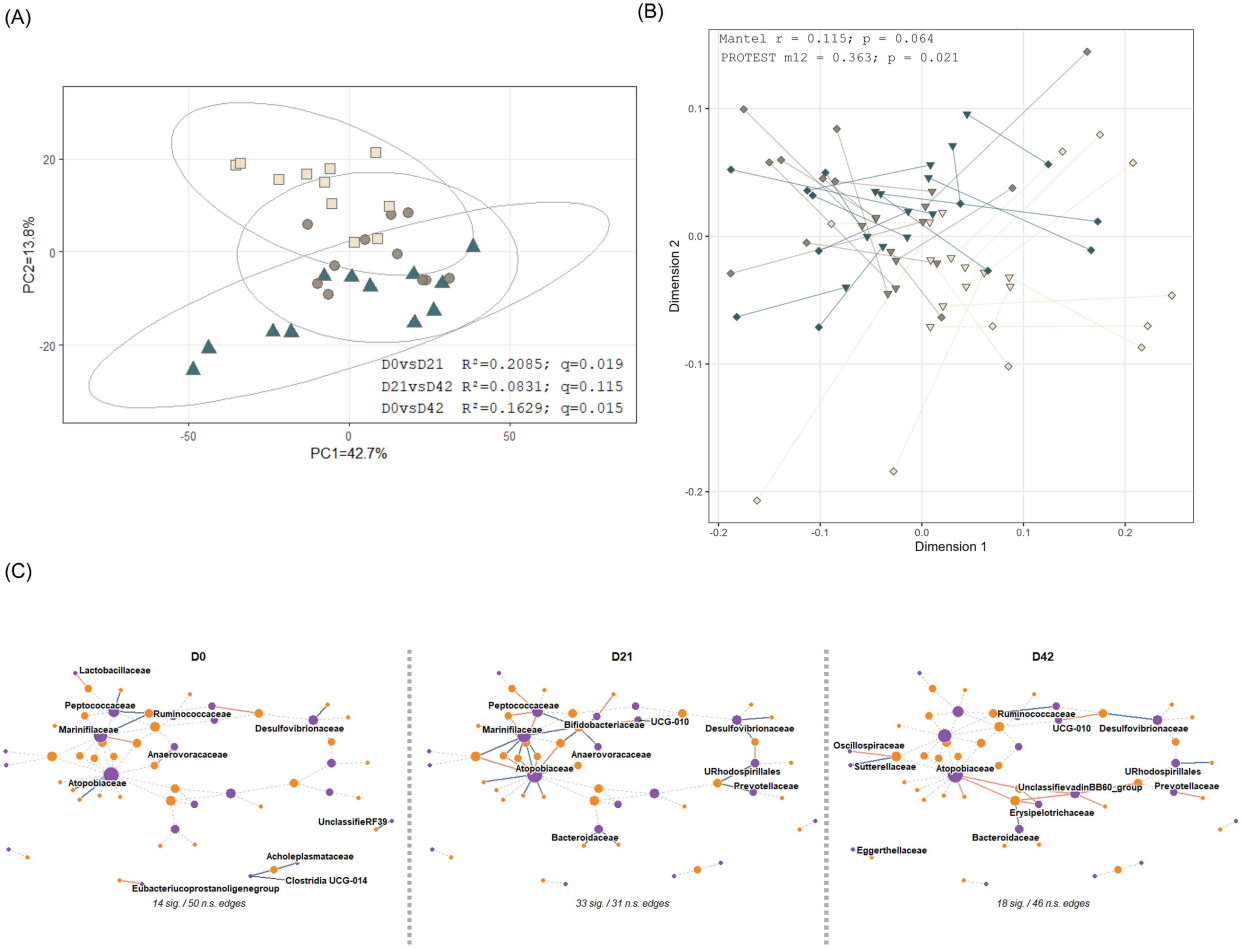
Longitudinal integration of the fecal metabolome and microbiota profiles. **(A)** Joint Principal Component Analysis (PCA) plot of fecal microbiome profiles at D0 (beige squares), D21 (brown circles), and D42 (dark cyan triangles). Each point represents cage-level biological units sampled longitudinally (*n =* 10–12). Shaded ellipses represent 95% confidence intervals for each timepoint. Statistical significance was assessed using pairwise PERMANOVA with FDR correction was applied to Euclidean distance matrices, which indicated significant differences between timepoints (*q* < 0.05). **(B)** Procrustes analysis visualizing the overall congruency between fecal metabolomic (triangle configuration) and microbiota (diamond configuration) datasets across all samples and timepoints (beige for D0, brown for D21, and dark cyan for D42) (*n =* 10–12). Points representing paired samples are connected by lines, where shorter lines indicate stronger congruency. Global agreement between datasets is shown. **(C)** Multi-omic correlation networks for D0, D21, and D42 using DIABLO for feature selection and Spearman as correlation method. Purple nodes represent microbial families and orange nodes represent detected metabolomic features. Blue edges indicate significant negative correlations, and red edges indicate significant positive correlations. Edge counts (significant vs. non-significant) are shown below each network. Bacterial family names are presented for those with statistically significant correlations.

Joint Principal Component Analysis (PCA) of the combined datasets revealed distinct clustering patterns, with D0 samples well-separated from D21 and D42 samples along the primary axis (Figure 5A). Pairwise PERMANOVA confirmed that the integrated metabolome-microbiota state at D21 differed significantly (*R^2^* = 0.2085, *q* = 0.019). Although the transition from D21 to D42 showed a reduced effect size (*R^2^* = 0.0831, *q* = 0.115), the final state at D42 remained significantly distinct from the D0 baseline (*R^2^* = 0.1629, *q* = 0.015).

Global congruency between the fecal metabolome and microbiota datasets was evaluated using Procrustes analysis. Global Procrustes transformation demonstrated significant longitudinal coordination between the two omics layers across the assessed timepoints (PROTEST *m_12_* = 0.363, *p* = 0.021; Figure 5B), whereas the Mantel test showed a positive but non-significant correlation (*r* = 0.115; *p* = 0.064). The short connecting lines linking paired metabolome-microbiota configurations for several samples further support coherent, time-dependent covariation between the two datasets.

Pairwise longitudinal integration revealed that the most significant coordinated shift occurred between D0 and D21 (Mantel *r* = 0.167, *p* = 0.013, *q* = 0.039; PROTEST *m_12_* = 0.483, *p* = 0.023, *q* = 0.069; Table 1), while no significant correlation was observed in the transition from D21 to D42 (Mantel *r* = −0.101, *p* = 0.767, *q* = 0.767; PROTEST *m_12_* = 0.309, *p* = 0.407, *q* = 0.407; Table 1), or between the D0 and D42 states (Mantel *r* = 0.022, *p* = 0.066, *q* = 0.099; PROTEST *m_12_* = 0.383, p = 0.064, *q* = 0.096; Table 1). Coordination between microbiota and metabolome was strongest between D0 and D21, indicating that the largest concurrent shifts in composition and metabolic output occurred during early exposure.

**Table 1:** Statistical assessment of the longitudinal congruency between fecal metabolome and microbiota profiles.

Comparison	*N*	Mantel			Protest		
		*r*	p-raw	*q_FDR_*	*m_12_*	p-raw	*q_FDR_*
D0 vs. D21	10	0.167	0.013	0.039	0.483	0.023	0.069
D21 vs. D42	11	−0.101	0.767	0.767	0.309	0.407	0.407
D0 vs. D42	11	0.022	0.066	0.099	0.383	0.064	0.096

Notably, cross-sectional Procrustes analysis at each timepoint revealed that the two omics layers were not significantly coupled at D0, D21, or D42 (*p* > 0.05; Supplementary Figure S2). These findings indicate that the alignment between the two omics layers arises from longitudinal dynamics across the intervention timeframe rather than from strong associations within individual timepoints.

To further explore the structure of these cross-omic relationships at each timepoint, DIABLO-based multi-omic networks were built to link microbial families to metabolomic features (Figure 5C). Network density and composition differed markedly across time. At D0, a limited number of significant correlations (*n =* 14) were observed, forming sparse connections between bacterial families and metabolites. At D21, the network became substantially more interconnected, with a larger number of both positive and negative correlations (*n =* 33), suggesting enhanced coordination between microbial activity and metabolite production during early dietary exposure. Key hub taxa at D21 included Acholeplasmataceae, Atopobiaceae, and Bacteroidaceae, which exhibited multiple significant edges with fecal metabolomic features. At D42, the network reorganized again, showing a distinct set of microbial families linked to metabolomic signatures, consistent with longer-term adaptation of the gut ecosystem to the berry-enriched diet. Importantly, network edges represent statistical correlations rather than direct biological interactions. Across the three networks, only bacterial families with statistically significant associations were retained, highlighting key taxa contributing to multi-omic integration over time.

Together, these analyses demonstrate that the longitudinal monitoring of berry-supplemented cohort reveals not only individual shifts in the fecal microbiota and metabolome but also progressively strengthens and reshapes the functional relationships between them, revealing coordinated ecosystem-level adaptation across the 42-day intervention.

## 4 Discussion

The findings of this study support a two-phase model of microbiome adaptation to dietary (poly)phenols, characterized by an early reconfiguration phase followed by a stabilization. Accordingly, this longitudinal multi-omic analysis shows that a berry-enriched diet reshapes the murine fecal ecosystem in a time-dependent manner, with the largest changes occurring during the first 21 days of intervention, followed by a relatively attenuated phase thereafter. This pattern was consistent across the metabolome, the microbiota, and their integration. At the metabolomic level, both D21 and D42 were clearly separated from baseline, whereas the D21 to D42 shift was smaller in magnitude (Figures 1A,B). A similar pattern was observed in the microbiota, where beta diversity and family-level clustering showed a marked displacement from D0 to the post-intervention states, while D21 and D42 remained comparatively closer to each other (Figures 2C,E). The integrated analyses reinforced this interpretation, with the strongest microbiota-metabolome coordination detected during the D0 to D21 transition (Figures 5A–C). Together, these findings indicate that the major restructuring of the fecal ecosystem occurs within the first 21 days of berry supplementation, followed by a more stable phase thereafter. Overall, this behavior is consistent with ecological models of perturbation-response systems, in which initial exposure to a selective pressure is associated with a rapid community reconfiguration, followed by convergence towards a new functional state. In this context, berry-derived (poly)phenols may act as both substrates and selective modulators, shaping microbial competition and metabolic network reconfiguration.

The metabolomic data further suggest that this early adaptation primarily involves reprogramming of metabolite abundance within a largely conserved compositional framework. Although 49.1% of all detected features were shared across the three timepoints, a substantial number of features changed longitudinally, indicating that variations in the relative abundance patterns predominated rather than replacing the fecal metabolome (Figures 1C,D). This is consistent with prior evidence from berry interventions, as a substantial fraction of berry-derived (poly)phenols and associated plant components reaches the colon and undergoes extensive microbial biotransformation into lower-molecular-weight compounds that can further influence host and microbial metabolism (15). Such reciprocal interaction, in which dietary compounds shape the microbiota and its metabolic activity and microbial metabolism in turn modifies dietary substrates, is well established and supports the concept of a diet-microbe-metabolite feedback loop (11, 18).

One of the most notable early signals in our dataset was the enrichment of the putatively identified tryptophan pathway among metabolites altered between D0 and D21 (Figures 3B,C). The early enrichment of tryptophan-related pathways highlights a rapid engagement of host-microbiota co-metabolic axes during the initial adaptation phase. Tryptophan is a central metabolic node at the host-microbiota interface, with its catabolism distributed across a variety of biologically active molecules that play key roles in the gut and beyond, including neurotransmitters (e.g., serotonin), immune-modulating compounds (e.g., kynurenine, quinolinate) and microbial indoles (39). This is consistent with broader metabolomic evidence indicating that microbiota-dependent aromatic amino acid and indole-derived metabolites are linked to host physiological processes, including gut-brain communication (40), early disease predictors (41), and circadian-related effects (42). Because these pathways generate metabolites with immunoregulatory, barrier-associated, and neuroactive relevance, this early shift may indicate a transient reorientation of host-microbiota metabolic crosstalk in the gut rather than an isolated change in amino acid abundance. In this framework, D21 alterations could represent an early functional adaptation to the dietary intervention, with potential downstream consequences for intestinal homeostasis and gut-brain signaling (40, 43).

By D42, the metabolomic profile was more strongly associated with the putatively identified tyrosine-derived aromatic compounds, including metabolites linked to catecholamine metabolism (Figures 4B,C). Given that catecholamine biosynthesis is a branch of tyrosine metabolism (44), and that aromatic metabolites detected in fecal metabolomics may reflect both putative berry-derived (poly)phenol catabolites (phenolic acids and aromatic derivatives) and broader aromatic amino acid metabolism (45), this long-term pattern is best interpreted as a diet-associated reorganization of aromatic metabolic signatures within the gut ecosystem rather than evidence for a specific enzymatic mechanism. Consistent with this, high-coverage metabolomic analyses have shown that microbiota-sensitive fecal metabolite profiles include aromatic amino acids, indoles, and catecholamine-related compounds, supporting the biological relevance of these signatures beyond fecal chemical composition alone (40). This interpretation is consistent with the later-phase comparison, in which D21 and D42 still differed at the global level (Figure 1A) but no newly enriched metabolite pathways were detected (Supplementary Figure S1B), supporting the view that the late phase reflects continuation of the initial response rather than the emergence of a distinct metabolic program.

The microbiota data mirrored this two-stage response. Shannon diversity increased earlier, between D0 and D21, whereas Chao1 richness increased later, between D21 and D42 (Figures 2A,B), suggesting that initial exposure co-occurred with an alteration in the relative balance of the existing community and only later expanded the breadth of represented families. Beta diversity, core microbiota analysis, and family-level clustering all converged on the same broader conclusion: the berry-enriched diet preserved a stable backbone of shared taxa while redistributing a smaller set of transient or diet-responsive families (Figures 2C–E). This is consistent with previous evidence that (poly)phenol-rich diets modulate gut microbial communities in a context-dependent manner (46). Within this broader pattern, the strongest family-level shifts were concentrated in a limited number of abundant taxa. Prevotellaceae and Muribaculaceae decreased relative to baseline in the Bacteroidota-dominated cluster (Figures 3E, 4E), whereas Lachnospiraceae and Oscillospiraceae increased in the Bacillota-associated cluster (Figures 3F, 4F). Muribaculaceae are particularly relevant in mice because they are commonly associated with glycan utilization and broader carbohydrate metabolism (47). In contrast, Lachnospiraceae are recognized fermenters of complex polysaccharides and participants in gut cross-feeding networks (48). The later increase in Muribaculaceae between D21 and D42 (Supplementary Figure S1D), following an earlier decrease, further suggests that some berry-responsive taxa may follow dynamic trajectories over time, rather than a simple linear response.

The observed decrease in Akkermansiaceae following berry supplementation highlights the context-dependent nature of microbiota responses (Figures 2D, 3D, 4D). Although the role of *Akkermansia* is often considered beneficial (linked to improved metabolic health and gut barrier function) (49, 50), it is highly context dependent. While berry-derived (poly)phenol interventions have often been associated with increased *Akkermansia* abundance together with positive effects (e.g., enhanced mucus layer and metabolic benefits) (49, 50), increased *Akkermansia* has also been reported in some disease-associated microbiota signatures (51), including Parkinson’s disease (52). In the present study, however, this taxonomic group was reduced relative to baseline. This should also be interpreted considering taxonomic resolution, since most available evidence refers to the genus *Akkermansia*, particularly *A. muciniphila*, rather than to the Akkermansiaceae family. Given the low abundance and transient detection of Akkermansiaceae at baseline, this shift should not be overinterpreted in isolation. Rather, the present findings support the view that microbiota responses to berry-derived (poly)phenols are best understood at the community level, with the overall impact of the intervention reflecting selective ecosystem reorganization rather than the effect of any single taxon. The inferred functional data add a further layer to this interpretation. Both D21 and D42 inferred microbial functional profiles were characterized by lower representation of several nucleotide-related categories and higher representation of pathways linked to carbohydrate metabolism, glycan biosynthesis and metabolism, and secondary metabolites biosynthesis, transport and catabolism (Figures 3G, 4G). While these results are based on functional predictions rather than direct measurements, they are consistent with known effects of diet-derived (poly)phenol interventions that can modulate microbiota and functional potential. This longitudinal design in healthy mice delineates the timing and coordinated nature of these longitudinal reconfigurations, addressing gaps left by prior cross-sectional and disease-model studies (11, 50, 53).

The integrated multi-omic analysis further supported coordinated longitudinal variation in the microbiota and fecal metabolome, with the strongest correspondence between the two datasets observed during the early response to the berry-enriched diet (Figures 5B,C). Importantly, this coordination was more evident across the temporal trajectory of the intervention than within individual timepoints, as timepoint-specific Procrustes analyses were not significant (Supplementary Figure S2). Together, these findings indicate that the diet-associated relationship between the two omic layers is better captured during periods of active ecosystem reconfiguration, for example when newly available substrates and shifting microbial niches likely drive concurrent changes in community structure and metabolic output, rather than as a static association measurable at isolated sampling points. The absence of strong timepoint-specific concordance is also consistent with non-synchronous dynamics, in which compositional restructuring and metabolite turnover may occur on different timescales and/or with lagged responses. That distinction matters because diet-microbiome relationships are increasingly recognized as dynamic and dependent on recent dietary history, meaning that single-timepoint analyses can obscure how microbial and metabolic layers co-adapt over time. In that sense, the longitudinal design used here is a major strength of the study.

Several limitations should be considered when interpreting these findings. The use of a single ionization mode in the untargeted metabolomic approach may reduce the detection of metabolites preferentially ionized in positive mode, thereby limiting the overall metabolome coverage. Although the 16S dataset provided robust taxonomic information, its resolution at the family level, limited more precise taxon-to-function interpretation. Metabolite annotations remained putative, pathway enrichment analyses were inference-based rather than structurally confirmed, and the functional microbiota results were prediction-based rather than directly measured. In addition, because host physiological readouts were not integrated here, the present findings are best interpreted at the level of gut ecosystem remodeling rather than downstream systemic effects. Additionally, a primary design limitation is the absence of a parallel, time-matched standard chow control group to fully isolate the berry intervention from natural temporal drift or continuous adolescent maturation in our 6-week-old cohort, as age and temporal progression are well-documented independent drivers of microbiome composition in rodent models (25, 26, 54–56). Our datasets revealed a sharp, non-linear biphasic kinetic curve, characterized by an acute restructuring from Day 0 to Day 21 followed by a stabilization through Day 42, strongly implying that the dietary intervention overrode background chronological drift. Importantly, these findings suggest that the timing of sampling critically influences the interpretation of diet-microbiome interactions, as early and late responses capture fundamentally different ecosystem states.

Nonetheless, the longitudinal integration of microbiota and metabolomic data within the same experimental unit, the cages, provide a comprehensive and biologically grounded view of the response to berry supplementation. Taken together, our results support a model in which berry supplementation induces an early, coordinated reorganization of the fecal gut ecosystem, followed by a stabilization phase characterized by more selective adjustments. This response was reflected by coordinated changes in taxonomic composition, aromatic metabolite signatures, predicted carbohydrate and glycan-related microbial functions, and the temporal coupling between microbiota structure and metabolic output. Thus, the impact of the berry-enriched diet was not limited to compositional variation but extended to a broader reorganization of the functional landscape of the gut ecosystem.

## Data Availability

The datasets generated in this study can be found in the NCBI Sequence Read Archive (SRA), under the BioProject accession PRJNA1465209. The datasets analyzed can be found in the MetaboLights Database, under accession number MTBLS14779.
