# Supplementary figures and images for: Longitudinal integration of microbiota and metabolomics reveals (poly)phenols-driven gut ecosystem dynamics
Source: Front Nutr. 2026 Jul 13;13:1858875. doi: 10.3389/fnut.2026.1858875 (PMC13402386; doi:10.3389/fnut.2026.1858875)

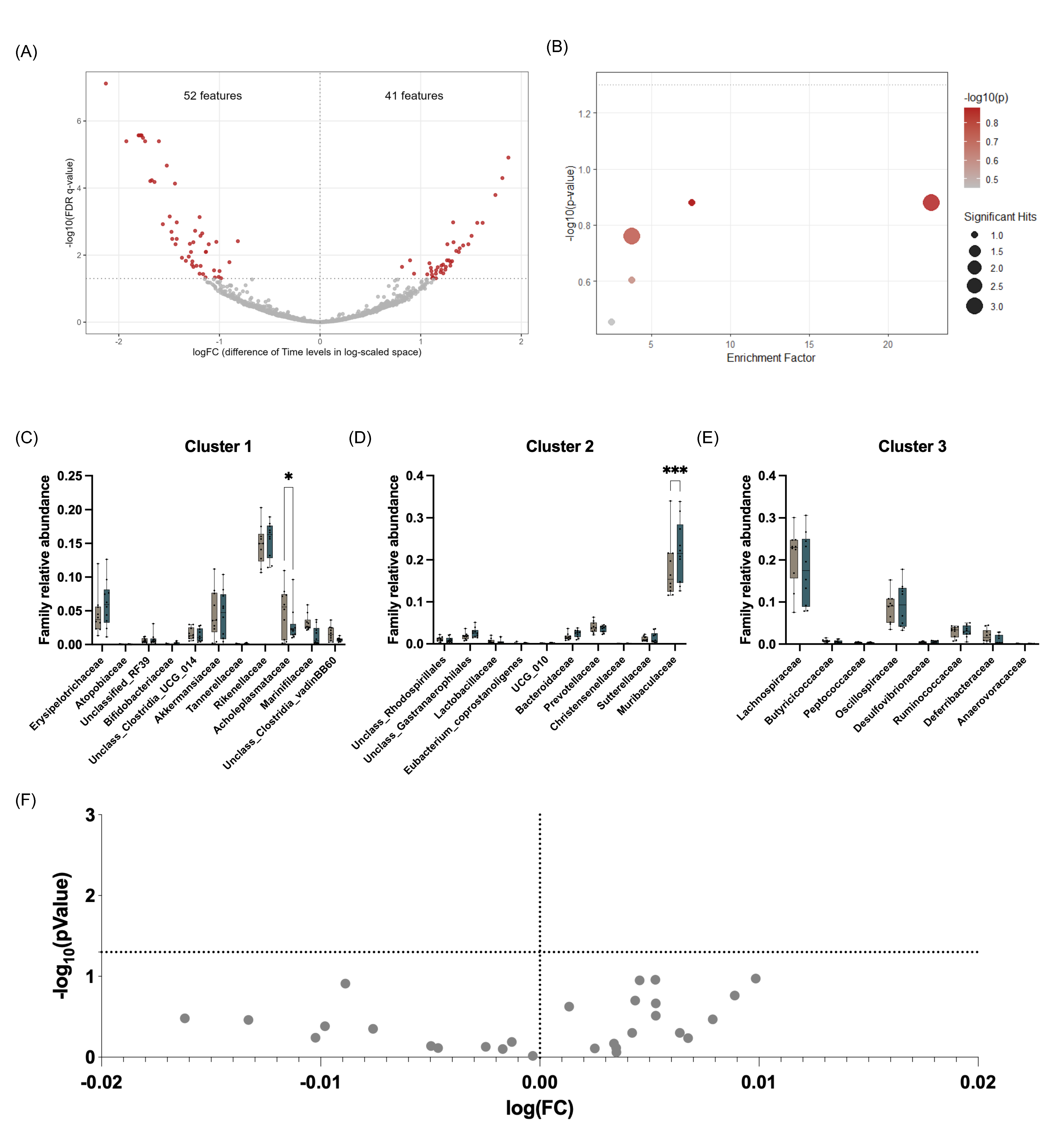

Supplement: Supplementary file 1 [file Image_1.TIFF]

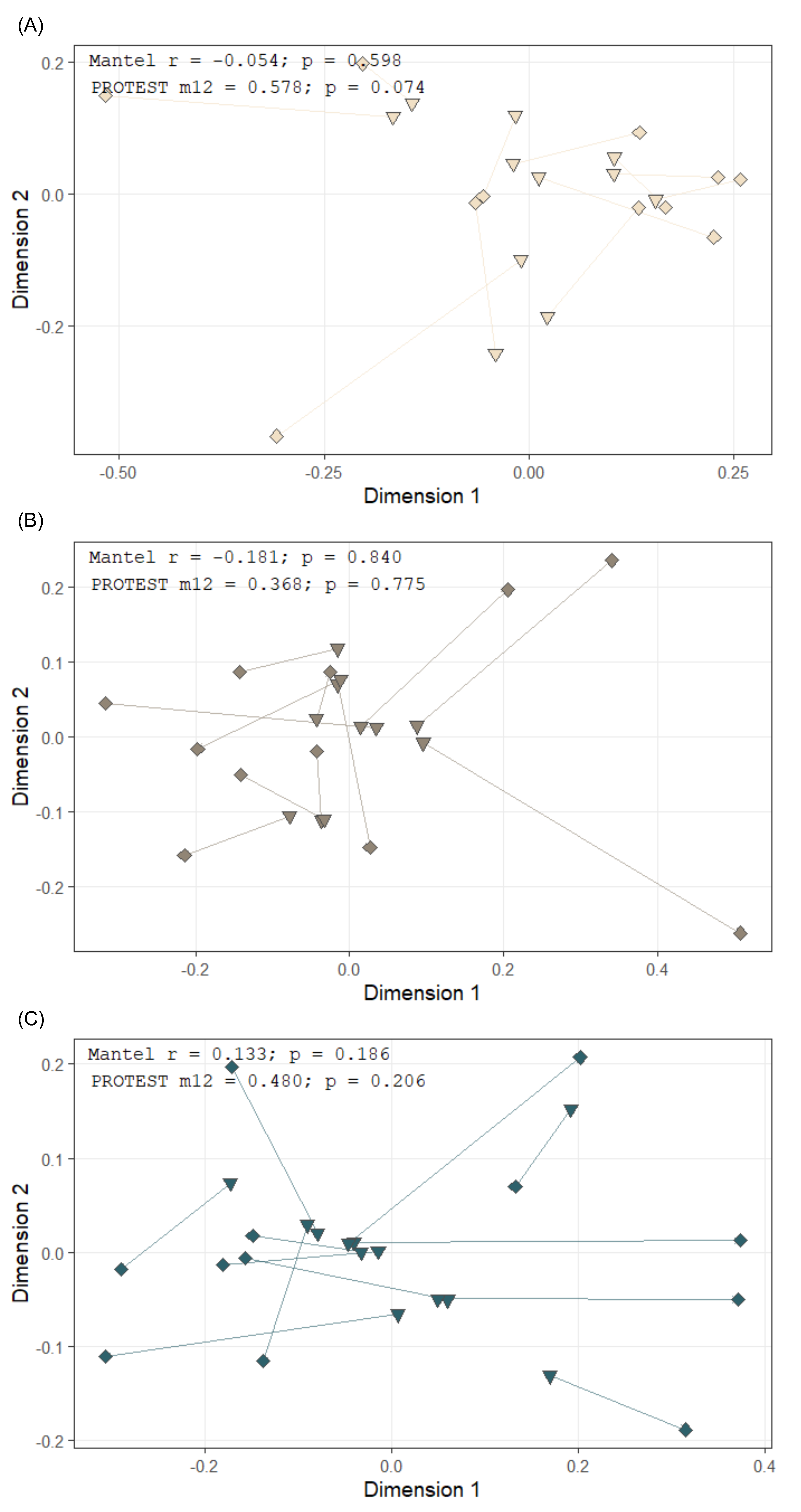

Supplement: Supplementary file 2 [file Image_2.TIFF]
