# Supplementary material for: Longitudinal integration of microbiota and metabolomics reveals (poly)phenols-driven gut ecosystem dynamics
Source: Front Nutr. 2026 Jul 13;13:1858875. doi: 10.3389/fnut.2026.1858875 (PMC13402386; doi:10.3389/fnut.2026.1858875)
Supplement: Supplementary file 3 [file Supplementary_file_1.DOCX]

**Longitudinal Integration of Microbiota and Metabolomics Reveals (Poly)phenols‑Driven Gut Ecosystem Dynamics**

Carlos Pita^1,2,#^, Catarina J. G. Pinto^1,2,3,4,#^, Rafael Carecho^2^, Natasa Loncarevic^1^, María Ángeles Ávila-Gálvez^2,5^, Juan Carlos Espín^5^, Antonio González-Sarrías^6^, David Berry^7^, Cláudia Nunes dos Santos^1,2,*^

^1^ iNOVA4Health, NOVA Institute Medical Systems Biology, NIMSB, Universidade Nova de Lisboa, 1099-085 Lisboa, Portugal

^2^ iNOVA4Health, NOVA Medical School | Faculdade de Ciências Médicas, NMS|FCM, Universidade Nova de Lisboa; Lisboa, Portugal.

^3^ IBMC, Instituto de Biologia Molecular e Celular, Universidade do Porto, Rua Alfredo Allen, 208, 4200-135, Porto, Portugal.

^4^ I3S, Instituto de Investigação e Inovação em Saúde, Universidade do Porto, Rua Alfredo Allen, 208, 4200-135, Porto, Portugal.

^5^ Laboratory of Food & Health, Group of Quality, Safety, and Bioactivity of Plant Foods, CEBAS-CSIC, Campus de Espinardo, 30100 Murcia, Spain.

^6^ Group of Quality, Safety, and Bioactivity of Plant Foods, CEBAS-CSIC, Campus de Espinardo, 30100 Murcia, Spain.

^7^ Centre for Microbiology and Environmental Systems Science, Department of Microbiology and Ecosystem Science, Division of Microbial Ecology, University of Vienna, Djerassiplatz 1, 1030 Vienna, Austria.

***Correspondence:**claudia.nunes.santos@unl.pt

**# Authors contributed equally**

**Supplementary Information**

**Supplementary Materials and Methods**

**Determination of proximate composition**

The proximate composition of both standard and berry-enriched diets was determined based on the Association of Official Analytical Chemists (AOAC) standard analysis methods, as previously described (1). Water content was determined by drying at 103 ± 2 ºC and ash at 550 ± 50 ºC. Total nitrogen was measured via the Kjeldahl method, with crude protein calculated by multiplying nitrogen content by 6.25. Crude fat was extracted with petroleum ether using Soxhlet, and crude fibre was analysed using the Weende method. Carbohydrates were estimated by subtracting water, ash, protein, fat, and fibre from the total.

**HPLC-ESI-IT-MS/MS analysis of animal diets for phenolic compounds**

Freeze-dried samples of both the standard and berry-supplemented diets were analyzed for phenolic composition. Briefly, 150 mg of freeze-dried material was extracted with 10 mL of methanol:dimethyl sulfoxide:water (40:40:20, v/v/v) acidified with 0.1% HCl to minimize phenolic oxidation. Samples were vortexed for 2 min, sonicated in an ultrasonic bath for 20 min, and centrifuged at 4000 × g for 10 min. The supernatants were collected and filtered through 0.45 µm polyvinylidene difluoride (PVDF) filters prior to HPLC-MS/MS analysis.

Phenolic compounds were analyzed using an Agilent 1200 HPLC system equipped with a photodiode array detector (DAD) coupled to a Bruker Daltonik ion trap mass spectrometer. Separation was performed on a Pursuit XRs C18 reverse-phase column (250 × 4.0 mm, 5 µm). The mobile phases consisted of (A) water containing 1% (v/v) formic acid and (B) acetonitrile, delivered at a flow rate of 0.8 mL/min. The elution gradient was as follows: 0 min, 5% B; 0-15 min, 5-20% B; 15-30 min, 20-55% B; and 30-45 min, 55-90% B. Phase B was then returned to 5% over 3 min, followed by a 6 min re-equilibration step. The injection volume was 20 µL.

UV-Vis spectra were recorded between 200 and 600 nm to monitor absorbance profiles of phenolic compounds. Mass spectra were acquired in both negative and positive ionization modes to detect phenolic species and anthocyanins. Nitrogen was used as both nebulizing and drying gas, at 65 psi, 11 L/min, and 350 °C. The capillary voltage was set at 4000 V. Mass spectra were collected over an m/z range of 100-1100, with a target mass of 500 m/z. Auto-MS mode was used to obtain fragmentation patterns for compound identification. All samples were analyzed in triplicate.

Phenolic compounds were identified based on elution order, UV absorbance spectra, molecular ions, and MS/MS fragmentation patterns, and were confirmed with authentic standards when available (Supplementary Table S1 and Supplementary Table S2). Quantification was performed using external calibration curves generated with representative standards for each phenolic class. Anthocyanins were quantified as cyanidin-3-*O*-glucoside equivalents at 520 nm; ellagic acid derivatives as ellagic acid equivalents at 360 nm; flavan-3-ols as catechin equivalents at 280 nm; hydroxycinnamic acids as chlorogenic acid equivalents at 320 nm; and flavonols as quercetin-3-*O*-rutinoside (rutin) equivalents at 360 nm. Each calibration curve was applied to the corresponding phenolic class for quantification in the diet samples.

**Supplementary Tables**

**Table S1** - Proximate composition of the standard diet and the berry-enriched diet based on the standard methods of the Association of Official Analytical Chemists (AOAC).

|  | ***Units*** | **Standard diet** | **Berry-enriched diet** |
| --- | --- | --- | --- |
| **Energy** | kJ / g | 15.81 ± 0.17 | 15.35 ± 0.11 |
|  | kcal / g | 3.76 ± 0.04 | 3.65 ± 0.03 |
| **Water** | % | 5.14 ± 0.09 | 7.52 ± 0.05 |
| **Dry matter** |  | 94.86 ± 0.09 | 92.48 ± 0.04 |
| **Carbohydrates** | g/100g | 63.52 ± 0.58 | 62.27 ± 0.36 |
| **Proteins** |  | 18.47 ± 0.20 | 17.07 ± 0.06 |
| **Lipids** |  | 4.70 ± 0.02 | 4.51 ± 0.01 |
| **Fibres** |  | 3.07 ± 0.37 | 3.74 ± 0.39 |
| **Ashes** |  | 5.09 ± 0.06 | 4.89 ± 0.07 |

**Table S2.** Phenolic compounds quantified by HPLC-UV-IT in the berry-supplemented diet.

| **Compounds** | **RT**  **(min)** | ***m/z^+^*** | **MS/MS** | **λ_max_**  **(nm)** | **mg/100 g** |
| --- | --- | --- | --- | --- | --- |
| **Total *Anthocyanins:*** |  |  |  |  | **30.85 ± 3.59** |
| Delphinidin-*O*-galactoside | 12.10 | 465 | 303 | 524 | 1.22 ± 0.08 |
| Delphinidin-*O*-glucoside | 13.30 | 465 | 303 | 524 | 5.32 ± 0.93 |
| Cyanidin-*O*-galactoside | 14.00 | 449 | 285 | 522 | 7.18 ± 1.01 |
| Cyanidin-3-*O*-glucoside* | 15.20 | 449 | 285 | 518 | 10.58 ± 0.92 |
| Petunidin-*O*-glucoside | 16.00 | 479 | 317 | 526 | 1.12 ± 0.04 |
| Malvidin-*O*-glucoside | 17.40 | 493 | 331/315 | 520 | 3.57 ± 0.51 |
| Peonidin-*O*-glucoside | 18.90 | 463 | 301 | 526 | 1.86 ± 0.10 |
|  | | ***m/z^-^*** |  | | |
| **Total *Hydroxycinnamic acids:*** |  |  |  |  | **6.72 ± 0.12** |
| Chlorogenic acid* | 14.60 | 353 | 191 | 296/312 | 3.05 ± 0.02 |
| Di-*O*-caffeoylquinic acid | 19.70 | 515 | 353/335 | 298/326 | 2.10 ± 0.07 |
| Caffeic acid | 23.90 | 179 | - | 296/322 | 1.57 ± 0.03 |
|  | | | | | |
| **Total *Flavon-3-ols*** |  |  |  |  | **8.69 ± 1.54** |
| Catechin* | 17.30 | 289 | 245 | 280 | 8.69 ± 1.54 |
|  | | | | | |
| **Total *Ellagic acid derivatives*** |  |  |  |  | **11.92 ± 2.56** |
| Ellagic acid* | 22.90 | 301 | 256/184 | 254/360 | 11.92 ± 2.56 |
|  | | | | | |
| **Total *Flavonols*** |  |  |  |  | **3.14 ± 0.66** |
| Myricetin-*O*-glucoside | 20.10 | 479 | 316 | 258/362 | 0.35 ± 0.01 |
| Quercetin-3-*O*-rutinoside (rutin)* | 22.00 | 609 | 301 | 254/350 | 1.29 ± 0.31 |
| Quercetin-*O*-arabinoside | 24.70 | 433 | 301 | 248/348 | 0.66 ± 0.19 |
| Quercetin-*O*-rhamnoside | 25.70 | 447 | 301 | 248/350 | 0.84 ± 0.15 |

***Quantified with their authentical standards.**

**Table S3.** Phenolic compounds quantified by HPLC-UV-IT in the standard diet.

| **Compounds** | **RT**  **(min)** | ***m/z^-^*** | **MS/MS** | **λ_max_**  **(nm)** | **mg/100 g** |
| --- | --- | --- | --- | --- | --- |
| **Total *Hydroxycinnamic acids:*** |  |  |  |  | **3.3 ± 0.10** |
| Di-*O*-caffeoylquinic acid | 19.70 | 515 | 353/335 | 298/326 | 2.75 ± 0.09 |
| Caffeic acid | 23.90 | 179 | - | 296/322 | 0.55 ± 0.10 |

**Table S4.** Pathway enrichment analysis between D0 and D21 in negative ionization mode, using the Mummichog algorithm and the *Mus musculus* KEGG library. Analysis was performed in MetaboAnalyst 6.0.

|  | **Pathway total** | **Hits.total** | **Hits.sig** | **Expected** | **P(Fisher)** | **P(EASE)** | **P(Gamma)** | **Emp.Hits** | **Empirical** | **AdjP.Fisher** | **AdjP.EASE** | **AdjP.Gamma** |
| --- | --- | --- | --- | --- | --- | --- | --- | --- | --- | --- | --- | --- |
| **Tryptophan metabolism** | 41 | 5 | 5 | 3.3833 | 0.0040413 | 0.034281 | 0.0020687 | 0 | 0 | 0.1050738 | 0.891306 | 0.0537862 |
| **Ascorbate and aldarate metabolism** | 9 | 5 | 5 | 2.4167 | 0.15656 | 0.43548 | 0.0035777 | 16 | 0.16 | 1 | 1 | 0.0894425 |
| **Linoleic acid metabolism** | 4 | 1 | 1 | 0.96667 | 0.22938 | 0.71977 | 0.0046778 | 22 | 0.22 | 1 | 1 | 0.1122672 |
| **Vitamin B6 metabolism** | 9 | 2 | 2 | 0.96667 | 0.22938 | 0.71977 | 0.0046778 | 9 | 0.09 | 1 | 1 | 0.1122672 |
| **Metabolism of xenobiotics by cytochrome P450** | 64 | 3 | 3 | 0.96667 | 0.22938 | 0.71977 | 0.0046778 | 22 | 0.22 | 1 | 1 | 0.1122672 |
| **Drug metabolism - cytochrome P450** | 21 | 2 | 2 | 0.96667 | 0.22938 | 0.71977 | 0.0046778 | 20 | 0.2 | 1 | 1 | 0.1122672 |
| **Tyrosine metabolism** | 42 | 11 | 8 | 3.8667 | 0.31555 | 0.56734 | 0.0064678 | 24 | 0.24 | 1 | 1 | 0.129356 |
| **Phenylalanine, tyrosine and tryptophan biosynthesis** | 4 | 2 | 1 | 1.45 | 0.47458 | 0.85506 | 0.012064 | 27 | 0.27 | 1 | 1 | 0.229216 |
| **Inositol phosphate metabolism** | 21 | 2 | 2 | 1.45 | 0.47458 | 0.85506 | 0.012064 | 31 | 0.31 | 1 | 1 | 0.229216 |
| **Glycolysis or Gluconeogenesis** | 23 | 2 | 2 | 0.48333 | 0.48333 | 1 | 0.012501 | 32 | 0.32 | 1 | 1 | 0.229216 |
| **Fructose and mannose metabolism** | 18 | 3 | 3 | 0.48333 | 0.48333 | 1 | 0.012501 | 32 | 0.32 | 1 | 1 | 0.229216 |
| **Galactose metabolism** | 27 | 7 | 7 | 0.48333 | 0.48333 | 1 | 0.012501 | 32 | 0.32 | 1 | 1 | 0.229216 |
| **Steroid hormone biosynthesis** | 79 | 3 | 3 | 0.48333 | 0.48333 | 1 | 0.012501 | 55 | 0.55 | 1 | 1 | 0.229216 |
| **Caffeine metabolism** | 12 | 2 | 2 | 0.48333 | 0.48333 | 1 | 0.012501 | 31 | 0.31 | 1 | 1 | 0.229216 |
| **Taurine and hypotaurine metabolism** | 8 | 1 | 1 | 0.48333 | 0.48333 | 1 | 0.012501 | 22 | 0.22 | 1 | 1 | 0.229216 |
| **Starch and sucrose metabolism** | 11 | 2 | 2 | 0.48333 | 0.48333 | 1 | 0.012501 | 32 | 0.32 | 1 | 1 | 0.229216 |
| **Arachidonic acid metabolism** | 42 | 1 | 1 | 0.48333 | 0.48333 | 1 | 0.012501 | 27 | 0.27 | 1 | 1 | 0.229216 |
| **Biosynthesis of unsaturated fatty acids** | 36 | 1 | 1 | 0.48333 | 0.48333 | 1 | 0.012501 | 27 | 0.27 | 1 | 1 | 0.229216 |
| **Phenylalanine metabolism** | 10 | 3 | 1 | 1.9333 | 0.66815 | 0.92626 | 0.027669 | 48 | 0.48 | 1 | 1 | 0.229216 |
| **Citrate cycle (TCA cycle)** | 16 | 3 | 2 | 0.96667 | 0.73729 | 1 | 0.038464 | 57 | 0.57 | 1 | 1 | 0.269248 |
| **Ubiquinone and other terpenoid-quinone biosynthesis** | 7 | 2 | 1 | 0.96667 | 0.73729 | 1 | 0.038464 | 54 | 0.54 | 1 | 1 | 0.269248 |
| **Pentose and glucuronate interconversions** | 18 | 3 | 2 | 1.45 | 0.86864 | 1 | 0.079771 | 86 | 0.86 | 1 | 1 | 0.398855 |
| **Amino sugar and nucleotide sugar metabolism** | 39 | 8 | 4 | 1.45 | 0.86864 | 1 | 0.079771 | 67 | 0.67 | 1 | 1 | 0.398855 |
| **Glyoxylate and dicarboxylate metabolism** | 31 | 3 | 2 | 1.45 | 0.86864 | 1 | 0.079771 | 61 | 0.61 | 1 | 1 | 0.398855 |
| **Alanine, aspartate and glutamate metabolism** | 28 | 4 | 1 | 2.4167 | 0.96889 | 1 | 0.18725 | 94 | 0.94 | 1 | 1 | 0.398855 |
| **Purine metabolism** | 71 | 5 | 1 | 2.9 | 0.98529 | 1 | 0.24435 | 99 | 0.99 | 1 | 1 | 0.398855 |

**Table S5.** Pathway enrichment analysis between D21 and D42 in negative ionization mode, using Mummichog algorithm and the *Mus musculus* KEGG library. Analysis was performed in MetaboAnalyst 6.0.

|  | **Pathway total** | **Hits.total** | **Hits.sig** | **Expected** | **P(Fisher)** | **P(EASE)** | **P(Gamma)** | **Emp.Hits** | **Empirical** | **AdjP.Fisher** | **AdjP.EASE** |
| --- | --- | --- | --- | --- | --- | --- | --- | --- | --- | --- | --- |
| **Primary bile acid biosynthesis** | 46 | 1 | 1 | 0.13208 | 0.13208 | 1 | 0.0010936 | 2 | 0.02 | 0.92456 | 1 |
| **Steroid hormone biosynthesis** | 79 | 3 | 3 | 0.13208 | 0.13208 | 1 | 0.0010936 | 13 | 0.13 | 0.92456 | 1 |
| **Arachidonic acid metabolism** | 42 | 1 | 1 | 0.13208 | 0.13208 | 1 | 0.0010936 | 9 | 0.09 | 0.92456 | 1 |
| **Biosynthesis of unsaturated fatty acids** | 36 | 1 | 1 | 0.13208 | 0.13208 | 1 | 0.0010936 | 9 | 0.09 | 0.92456 | 1 |
| **Tryptophan metabolism** | 41 | 6 | 3 | 0.79245 | 0.17403 | 0.53228 | 0.0012966 | 9 | 0.09 | 0.92456 | 1 |
| **Metabolism of xenobiotics by cytochrome P450** | 64 | 2 | 1 | 0.26415 | 0.24891 | 1 | 0.0017646 | 13 | 0.13 | 0.92456 | 1 |
| **Amino sugar and nucleotide sugar metabolism** | 39 | 8 | 1 | 0.39623 | 0.352 | 1 | 0.0027257 | 21 | 0.21 | 0.92456 | 1 |

**Table S6.** Pathway enrichment analysis between D0 and D42 in negative ionization mode, using Mummichog algorithm and the *Mus musculus* KEGG library. Analysis was performed in MetaboAnalyst 6.0.

|  | **Pathway total** | **Hits.total** | **Hits.sig** | **Expected** | **P(Fisher)** | **P(EASE)** | **P(Gamma)** | **Emp.Hits** | **Empirical** | **AdjP.Fisher** | **AdjP.EASE** | **AdjP.Gamma** |
| --- | --- | --- | --- | --- | --- | --- | --- | --- | --- | --- | --- | --- |
| **Tyrosine metabolism** | 42 | 12 | 11 | 3.0556 | 0.00077812 | 0.0050285 | 0.004324 | 0 | 0 | 0.0155624 | 0.10057 | 0.08648 |
| **Arachidonic acid metabolism** | 42 | 3 | 2 | 1.2222 | 0.081956 | 0.33201 | 0.0055644 | 6 | 0.06 | 1 | 1 | 0.1057236 |
| **Biosynthesis of unsaturated fatty acids** | 36 | 1 | 1 | 0.61111 | 0.090376 | 0.50117 | 0.0057133 | 0 | 0 | 1 | 1 | 0.1057236 |
| **Linoleic acid metabolism** | 4 | 1 | 1 | 0.91667 | 0.21948 | 0.65082 | 0.0086213 | 14 | 0.14 | 1 | 1 | 0.1465621 |
| **Glycolysis or Gluconeogenesis** | 23 | 2 | 2 | 0.30556 | 0.30556 | 1 | 0.011435 | 0 | 0 | 1 | 1 | 0.18296 |
| **Fructose and mannose metabolism** | 18 | 3 | 3 | 0.30556 | 0.30556 | 1 | 0.011435 | 0 | 0 | 1 | 1 | 0.18296 |
| **Taurine and hypotaurine metabolism** | 8 | 1 | 1 | 0.30556 | 0.30556 | 1 | 0.011435 | 0 | 0 | 1 | 1 | 0.18296 |
| **Starch and sucrose metabolism** | 11 | 2 | 2 | 0.30556 | 0.30556 | 1 | 0.011435 | 0 | 0 | 1 | 1 | 0.18296 |
| **Glycerophospholipid metabolism** | 13 | 1 | 1 | 0.30556 | 0.30556 | 1 | 0.011435 | 0 | 0 | 1 | 1 | 0.18296 |
| **Phenylalanine metabolism** | 10 | 3 | 2 | 1.2222 | 0.35701 | 0.75709 | 0.013589 | 15 | 0.15 | 1 | 1 | 0.18296 |
| **Tryptophan metabolism** | 41 | 9 | 3 | 3.0556 | 0.35989 | 0.60764 | 0.013722 | 41 | 0.41 | 1 | 1 | 0.18296 |
| **Galactose metabolism** | 27 | 9 | 7 | 0.61111 | 0.52074 | 1 | 0.024129 | 21 | 0.21 | 1 | 1 | 0.217161 |
| **Ubiquinone and other terpenoid-quinone biosynthesis** | 7 | 2 | 1 | 0.61111 | 0.52074 | 1 | 0.024129 | 28 | 0.28 | 1 | 1 | 0.217161 |
| **Valine, leucine and isoleucine biosynthesis** | 8 | 4 | 2 | 0.61111 | 0.52074 | 1 | 0.024129 | 19 | 0.19 | 1 | 1 | 0.217161 |
| **Amino sugar and nucleotide sugar metabolism** | 39 | 5 | 4 | 0.61111 | 0.52074 | 1 | 0.024129 | 32 | 0.32 | 1 | 1 | 0.217161 |
| **Inositol phosphate metabolism** | 21 | 2 | 1 | 0.61111 | 0.52074 | 1 | 0.024129 | 27 | 0.27 | 1 | 1 | 0.217161 |
| **Vitamin B6 metabolism** | 9 | 2 | 1 | 0.61111 | 0.52074 | 1 | 0.024129 | 20 | 0.2 | 1 | 1 | 0.217161 |
| **Valine, leucine and isoleucine degradation** | 35 | 6 | 2 | 0.91667 | 0.67136 | 1 | 0.043052 | 41 | 0.41 | 1 | 1 | 0.217161 |
| **Phenylalanine, tyrosine and tryptophan biosynthesis** | 4 | 2 | 1 | 0.91667 | 0.67136 | 1 | 0.043052 | 36 | 0.36 | 1 | 1 | 0.217161 |
| **Ascorbate and aldarate metabolism** | 9 | 4 | 1 | 1.2222 | 0.77614 | 1 | 0.067855 | 66 | 0.66 | 1 | 1 | 0.217161 |

**References**

1. Gomes, Oudot, Macià, Foito, Carregosa, Stewart, Van de Wiele, Berry, Motilva, Brenner, et al. Berry-Enriched Diet in Salt-Sensitive Hypertensive Rats: Metabolic Fate of (Poly)Phenols and the Role of Gut Microbiota. *Nutrients* (2019) 11:2634. doi: 10.3390/nu11112634
